# Navigating beyond “here & now” affordances—on sensorimotor maturation and “false belief” performance

**DOI:** 10.3389/fpsyg.2014.01433

**Published:** 2014-12-15

**Authors:** Maria Brincker

**Affiliations:** Department of Philosophy, University of Massachusetts BostonBoston, MA, USA

**Keywords:** affordances, false belief test, metacognition, sensorimotor priors, developmental psychology, embodied cognition, theory of mind, social cognition

## Abstract

How and when do we learn to understand other people's perspectives and possibly divergent beliefs? This question has elicited much theoretical and empirical research. A puzzling finding has been that toddlers perform well on so-called implicit false belief (FB) tasks but do not show such capacities on traditional explicit FB tasks. I propose a navigational approach, which offers a hitherto ignored way of making sense of the seemingly contradictory results. The proposal involves a distinction between how we navigate FBs as they relate to (1) our current affordances (here & now navigation) as opposed to (2) presently non-actual relations, where we need to leave our concrete embodied/situated viewpoint (counterfactual navigation). It is proposed that whereas toddlers seem able to understand FBs in their current affordance space, they do not yet possess the resources to navigate in abstraction from such concrete affordances, which explicit FB tests seem to require. It is hypothesized that counterfactual navigation depends on the development of “sensorimotor priors,” i.e., statistical expectations of own kinesthetic re-afference, which evidence now suggests matures around age four, consistent with core findings of explicit FB performance.

## False belief tests and conflicting explicit and implicit findings

The question of how and when children learn to understand other people's beliefs and perspectives has long been an object of study and philosophical debate. Many experimental paradigms use false belief (FB) scenarios to test this development. Typical FB paradigms set up a discrepancy between a test subject's accurate information about a scenario and a divergent perspective, which then is used to probe whether the false perceptive of the other is taken into account. Interestingly so-called “explicit” and “implicit” categories of FB tests each consistently point to very different minimal ages for the development of FB abilities.

Explicit FB tests can be exemplified by the Sally-Anne task (Baron-Cohen et al., 1985), which involves a presented story with two protagonists Sally and Anne. The FB discrepancy is set up as the test subject watches that Sally puts a marble in location A and leaves, whereupon Anne unbeknownst to Sally transfers the marble to location B. The experimenter/story-teller then prompts the test subject to anticipate an action by the mislead Sally. e.g., “Where do you think Sally *would* look for her toy?” Researchers have overwhelmingly found that typically developing (TD) children generally do not “pass” this kind of FB test before about age four or older. Younger children have a strong tendency to suggest the current toy-location rather than where Sally left it (Wellman et al., 2001). If one assumes the test tracks abilities to understand “beliefs,” then results indicate that TD kids cannot handle others' beliefs before age four. Accordingly, failing to produce the correct answer has been interpreted as revealing either a categorical inability to understand minds (Baron-Cohen, 1995), or performance difficulties with linguistic aspects and/or with prioritizing and using FB information executively over current factual information (Moses et al., 2005; Carruthers, 2013).

Conclusions of relative “mind-blindness” in toddlers have been challenged by various so-called “implicit” experimental paradigms, showing that children seem to track and form FB expectations much earlier. These studies use non-verbal/active participation FB tasks, relying on either looking-time (Onishi and Baillargeon, 2005), communicative reference (Southgate et al., 2010) or helping paradigms (Buttelmann et al., 2009; Knudsen and Liszkowski, 2010, 2012). These paradigms do not explicitly ask the child about *expected actions of another agent*, but rather measures whether their *own action selections vary* significantly with respectively true/false belief conditions of observed others. e.g., in Buttelmann et al.'s helping paradigm the test subject is invited to open either of two boxes given a manipulation where, during the crucial toy transfer, an experimenter is observed either staying in the room (true belief) or leaving (FB). The remarkable finding was that even 18 months-olds varied their box choice significantly with the manipulation of the experimenters “belief scenario.” Thus, the results suggested that toddlers might indeed understand FBs. Looking-time paradigms indicate FB understanding perhaps as early as 9–15 months (Baillargeon et al., 2010; see also Reddy, 2008), but I focus uniquely on the helping paradigm and the specific puzzle of why 18–30 month-old toddlers appropriately vary their behavior according to perceived FBs (and also handle object permanence and pretense play), and yet consistently fail Sally-Anne style FB tests.

In addition to the age disparities between Sally-Anne and the helping-paradigm FB findings, the labels of implicit/explicit and their core experimental differences are not easily conceptualized, as discussed by Carruthers (2013). Existing theories often distinguish *information types* of FB tasks; i.e., mental, counterfactual, linguistic, conceptual, representational, etc. and theorize the *domain-specific* or *domain-general capacities* needed to handle these kinds of information. Further, debates often center on whether the observed infant/toddler capacities should be interpreted as based on mindreading or on behavioral rules (Knudsen and Liszkowski, 2010; Perner, 2010; Carruthers, 2013).

This perspective article points to an alternative framework for theorizing the Sally-Anne and helping-paradigm and the developmental processes underlying the discrepancy of their findings. Given the short format I do not argue against any existing theory, but rather propose that a sensorimotor maturation-based explanation would expand the existing interpretive possibility space, as mental processes are modeled differently than current categorizations imply.

The core proposal is that the age discrepancy of FB findings might be rooted in sensorimotor maturation processes taking place around age four, as these might ground the relevant cognitive developments documented at this age. It is suggested that the helping and Sally-Anne paradigms might require different kinds of “navigation,” which again depends on different levels of sensorimotor maturation. More precisely a distinction is drawn between the ability to navigate presently available “here & now” perceptual space vs. navigating an imagined, remembered or otherwise currently counterfactual bodily space. The latter ability to “navigate beyond the here & now” is hypothesized to depend on late-developing predictable movement variations, and further that such abilities support successful Sally-Anne FB performance. By contrast it is proposed that helping paradigm FB tasks are based on the child's understanding of the “here & now” social affordance space and thus can be navigated without this aspect of sensorimotor maturation. To explain this unexplored possibility and theorize the distinction between here & now and counterfactual navigation, we need to look to neuroscientific evidence for (1) affordance tracking and decision-making, (2) default systems and self-projection and lastly (3) the maturation of sensorimotor priors.

## Decision-making and navigation of “here & now” affordances

The idea that we track multiple affordances, i.e., perceived action possibilities in the “engageable” space around us, is not new (Gibson, 1977). Recently, the affordance notion has attracted renewed attention, through theories (Heft, 2003; Gallagher, 2005; Rietveld, 2008; De Jaegher et al., 2010), but also via neuroscientific discoveries about cortical fronto-parietal sensorimotor processes and massively parallel and dynamic circuits mediated by cortical and sub-cortical circuits (Cisek and Kalaska, 2010) and advances in e.g., robotics (Horton et al., 2012) where these kinds of ecological agent-environment relations have begun to replace traditional input-output representational frameworks. Cisek and Kalaska point to findings that sensorimotor processes are engaged early, in parallel and support not only action execution but also decision-making and action selection between multiple tracked options. They argue these findings of early and parallel affordance tracking are inconsistent with modular input-output information processing frameworks. Further, mirror neuron research has shown that we track object affordances as they relate to perceived others, as well as complex and dynamic social affordances between self and other (Casile et al., 2011; Sartori et al., 2012).

Thus, in opposition to classic notions of the mind as entirely hidden or “sandwiched” between action and perception (Hurley, 2001), a relational affordance story lets decision-making processes partially reveal themselves through not only actual but possible engagements with our environment. On a theoretical level such findings complicate our notion of social perception, as we see not only the actual behaviors of others, but their potential and afforded action targets, and how these relate to our own current action in overall shared affordance space. The key is that affordances alert to potential outcomes as they relate to actual objects and agents in the spatial environment.

Carruthers (2013) hypothesizes that FB abilities require a “domain specific mindreading module” (tracking other's goals, beliefs etc.), domain general planning, and decision-making abilities (own action selection) plus belief attribution processes. But the question is if we need to postulate a separate “mindreading module” and attribution processes for FB understanding. If sensorimotor processes ground a complex and dynamic tracking of current affordance relations of self and other (Trevarthen, 1979; De Jaegher and Di Paolo, 2007; Gallagher, 2012), then might we not oftentimes understand goals and FBs of interaction partners though this tracking of their actions and affordances and how they differ from and dynamically modulate our own?

Fronto-parietal processes have been found to support not only action planning and decision-making but also perception of the affordances of others. Notably these circuits show complex and dynamic properties, as affordances can be social, visible or hidden (e.g., Umilta et al., 2001). Further, the complexities of our context relations undergo various crucial maturation processes, particularly in the first years of life as evidenced by “A-not-B-error” (Smith and Thelen, 2003) and pretense play studies (Leslie, 1987) etc. For our present purposes we should note that affordances and thus action planning of 2–3 year-old toddlers is not necessarily restricted to what is presently *sensed*, but rather to that which offers our spatially situated bodies sensorimotor engagement. Thus, the door behind us might still be tracked as an afforded “escape-route” even if currently unseen. Similarly, a pretend banana, which is not actually there, can–as spatially actable—be part of the shared affordance space and must comply with certain rules of engagement. Research on mirror neuron circuits also indicates that others' actions and affordances are dynamically integrated into this affordance space understanding (Caggiano et al., 2009; Sartori et al., 2012). The “here & now” affordance space might thus contain counterfactual and prospective teleological *elements*—such as unseen and pretense affordances or others' falsely maintained affordances—as long as these are placed in relation to embodied agents (Gibson, 1977; Brincker, 2010, 2012). A source of complexity is that limits to the current affordance space are fluid, but it is an empirical question how far from our current position our skilled, cultured, and tool-enhanced bodies can track own and shared potentialities (Iriki, 2006).

In sum, the proposal is that we reach boxes, answer questions, and point to hidden marbles via a “here & now” affordance space, and further that FBs of others might, as long as they relate to the space we concretely inhabit, be understood, tracked, and engaged through our affordance space understanding.

Looking at the typical helping paradigms, they allow the child to incorporate relevant past and present perspectives of others into their present scenario, without requiring them to abstract from their embodied relation to it. e.g., 18/30 month-olds in the Buttelmann et al. study must integrate contrasting perspectives and “false beliefs” of others within their affordance space. However, they need not let go of their pragmatic relations to this current space to perform the task. Thus, it can be interpreted as “here & now” social navigation incorporating FB *content* in their actual body *space*.

In Sally-Anne style tests on the other hand, toddlers might be aware that Sally doesn't know and yet still not pass the test. Perhaps they even have extra difficulties not sharing the true location of the marble precisely *because* they know Sally doesn't know where it is. In navigational terms, passing the Sally-Anne test requires a child–beyond linguistic skills—to be able to handle the *conflicting pragmatic contexts* of the marble hunt story and the experimenter-meta-question. To pass this test one might need to (1) understand and remember Sally's perspective, but also—and this is where we seem to move beyond the here & now—(2) navigate the situation from her counterfactual vantage-point, which involves momentarily setting aside one's current position in relation to the marble, and finally (3) to return to the verbal prompt and respond to what Sally would have done (had we not been there to help her). Each of these subsequent aspects contribute to the complexity of this highly non-cooperative scenario where one in addition to *including the other's perspective*, also must *exclude one's own* embodied knowledge, and *navigate* both via the current affordance space and beyond it.

## Self-projection and navigating beyond the “here & now”

In contrast to here & now navigation, we sometimes—typically in our thoughts–engage in what we might call *counterfactual navigation*. i.e., when we place ourselves in remembered, imagined or otherwise not-actually-bodily-inhabited-spaces to use the resulting relational body-space understanding for various deliberations. The key is that it is the *relation* that is counterfactual, not the information or the objective existence of the space. Thus, such navigation goes beyond merely including *counterfactual information*, i.e., memories, perspectives or pretense objects, in our actually inhabited space and situated action choices. Rather it is about “placing oneself” and “making moves” via a space, which–although perhaps factually existing—is *pragmatically counterfactual*, and requires one to abstract from actual embodied relations to the current affordance space.

In terms of Sally-Anne tasks, one might thus be able to track Sally's FB in the current affordance space, but unable to plan a response from Sally's perspective and/or shift back and verbalize it in the prompt context, which requires one to ignore the current position of the marble as it relates to both oneself and Sally (see Bloom and German, 2000; Rubio-Fernández and Geurts, 2013 for Sally-Anne variations that probe some of these behavioral complexities and age limitations). Own prior beliefs and misperceived affordances might also under some conditions require counterfactual navigation, as in e.g., the Smarties box/ appearance-reality paradigms (Gopnik and Aslington, 1988). In short, task aspects of planning, deciding and inferring via remembered/projected scenarios can all be interpreted as involving navigating beyond the current affordance space, as one needs to relate to options, which cannot be interpreted through the sensorimotor affordance space that our situated bodies are actually dwelling in.

The idea of such counterfactual navigation differs from traditional modular, non-relational, and knowledge-focused aspects of “theory of mind” theories (e.g., Leslie et al., 2004; Carruthers, 2013), as no domain specific mindreading or ToM module is postulated. Rather the crucial distinction of the navigational hypothesis pertains not to the other mind *content per se* but rather to how it is presented, assessed or navigated by the understanding subject. Similarly, though there are parallels between the idea of counterfactual navigation and that of off-line simulation (e.g., Heal, 1996; Goldman, 2006), an important contrast is that counterfactual content or “pretense” can play a role in both kinds of navigation. Thus, specific overlaps and contrasts exist to aspects of traditional theory, ToMM etc.

Interestingly, the core cortical areas implicated in much social cognitive research of “theory of mind” have been found to overlap greatly with the default-mode network (Schilbach et al., 2008). The default mode network was precisely isolated due to its sustained activity in the absence of current external-directed attention and stimuli (Gusnard and Raichle, 2001), and has been interpreted as supporting various kinds of “self-projection” (Buckner and Carroll, 2007) or “internal mentation” (Andrews-Hanna, 2012), whether these pertain to social cognition, memory or future projection. In other words, it fits with a notion of navigation beyond the here & now.

Another line of social cognitive research that could be reinterpreted within a navigation framework is the role of the rTPJ in thinking about other minds. This cortical region has been consistently implicated in imaging studies and thus on a modular account proposed to support “theory of mind” (Saxe and Wexler, 2005). However, another possibility is that the region is important for *shifting* between here & now navigation and counterfactual navigation. This hypothesis would fit with broader evidence regarding the role of the rTPJ in attentional shifts (Mitchell, 2008).

These preliminary notes are meant simply to highlight that the navigation hypothesis throws a new light on existing imaging findings and most importantly is empirically tractable.

## Maturation of sensorimotor priors and counterfactual navigating

The distinction between these two kinds of navigation is proposed as a new interpretation of the age discrepancy of implicit vs. explicit FB tests. The idea is that what is maturing around age four might not be the ability to represent minds, non-current facts or that others have counterfactual beliefs. Rather the proposal is that a counterfactual use of one's embodied learning becomes possible, which again allows for ignoring “here & now” perspectival affordances and for acting on the basis of a navigation of counterfactual space. Toddlers are typically capable of remembering, making predictions, telling us what we don't know etc., and they actively do so in present contexts (Poulin-Dubois et al., 2007; Apperly and Butterfill, 2009). What the smaller children might not be able to do is pragmatically navigating counterfactual terrains. The hypothesis is thus that non-current information only can take on the needed affordance organization for action choice in relation to their current body.

But why would the counterfactual navigation not be available to toddlers? In other words, according to the navigation hypothesis what is the crucial maturation that happens around age four? Recent experimental data from Elizabeth Torres' sensorimotor lab points to a fascinating answer. She found that typically developing (TD) 3-year-olds do not yet have statistical predictability of temporal features of their limb movements (Torres et al., 2013). One might say that they do not yet have “sensorimotor priors” with respect to their own bodily movements and their ensuing re-afferent sensations (Von Holst and Mittelstaedt, 1950). Notably, Torres found that hand movement variations go through a crucial maturation precisely around age four. More specifically the variations are noisy and random in toddlers but then begin to show predictability and better signal-to-noise ratios in the 4 year-olds.

These are remarkable findings and their potential goes beyond this current project. The hypothesis that I would like to bring attention to here is that such “sensorimotor priors” can precisely be seen as a kind of predictable probabilistic body, an abstract body that we can “bring into” counterfactual scenarios and thus use to navigate and make decisions in spaces we do not stand in current embodied relations to. The idea is that only when we have established a reliable and predictable baseline expectation about our own re-afferent movements, can we use these hypothetically. In other words, sensorimotor priors as such embodied expectations might ground abstract navigational relations to non-present spaces, and thus allow us to navigate beyond the pragmatic relations of our actually situated bodies. Torres further found that this sensorimotor maturation does not follow the usual trajectory in individuals with autism—which suggests that they have to rely on their “here & now” body and world sensation in very different ways than TD children, and offers a new perspective on their social interaction and FB task difficulties (Brincker and Torres, 2013; Torres, 2013; Torres et al., 2013).

Thus, the proposal is that typical 2–3 year-olds can engage in elaborate pretense incorporating hypothetical content such as memories, fantasy objects and alternative perspectives in their present affordance space, relating to their bodies and sensorimotor capabilities. But they might not have the statistical body expectations needed to navigate counterfactual spaces “in their head” so to speak. The proposal is thus that the helping paradigm does indeed show early understanding of FBs, but not the ability to go beyond the current affordance relations to counterfactual spaces or mental navigation, which seems to be needed in the traditional Sally-Anne task. Under this framework, the development of sensorimotor priors around age four might transform some here & now social knowledge and interactions, but does not move the child from “mind-blindness” to “mindreading.”

### Conflict of interest statement

The author declares that the research was conducted in the absence of any commercial or financial relationships that could be construed as a potential conflict of interest.
